# A Manual of Blow-Pipe Analysis and Demonstrative Mineralogy

**Published:** 1866-11

**Authors:** 


A Manual of Blow-Pipe Analysis, and Determinative Mineralogy.

By WM. ELDERHERST, M.D., Prof. of Chemistry in the Rensselaer Polytechnic Institute. Third edition, revised and greatly enlarged. Philadelphia: T. ELLWOOD ZELL, Pub. 1866.

This is a small volume, and will be found interesting and valuable to all those who wish to study mineralogy and metallurgy.

